# The physiology of artificial hibernation

**Published:** 2015-09-30

**Authors:** Marcel C. Dirkes, Thomas M. van Gulik, Michal Heger

**Affiliations:** Department of Experimental Surgery, Academic Medical Center, University of Amsterdam, Amsterdam, the Netherlands

**Keywords:** anapyrexia, hypometabolic agents, hypometabolism, natural hibernation, torpor, thermoneutral zone, hypoxia, body temperature, thermal convection, Arrhenius law

## Abstract

Incomplete understanding of the mechanisms responsible for induction of hibernation prevent translation of natural hibernation to its artificial counterpart. To facilitate this translation, a model was developed that identifies the necessary physiological changes for induction of artificial hibernation. This model encompasses six essential components: metabolism (anabolism and catabolism), body temperature, thermoneutral zone, substrate, ambient temperature, and hibernation-inducing agents. The individual components are interrelated and collectively govern the induction and sustenance of a hypometabolic state. To illustrate the potential validity of this model, various pharmacological agents (hibernation induction trigger, delta-opioid, hydrogen sulfide, 5’-adenosine monophosphate, thyronamine, 2-deoxyglucose, magnesium) are described in terms of their influence on specific components of the model and corollary effects on metabolism.

**Relevance for patients:** The ultimate purpose of this model is to help expand the paradigm regarding the mechanisms of hibernation from a physiological perspective and to assist in translating this natural phenomenon to the clinical setting.

## Contents

1. Introduction (79)
2. A model for hypometabolic induction (80)
  2.1. Control of metabolism through temperature and substrate availability (80)
  2.2. Thermoregulation following a shift in the thermoneutral zone (80)
  2.3. Hypoxia-induced hypometabolism: aligning anabolism with catabolism (81)
  2.4. The effect of hypoxia-induced anapyrexia on thermoregulatory effectors (83)
  2.5. Pharmacological agents and induction of artificial hypometabolism (84)
    2.5.1. Hydrogen sulfide (84)
    2.5.2. Adenosine monophosphate (84)
    2.5.3. Thyronamines (86)
    2.5.4. Deoxyglucose (86)
    2.5.5. Delta-opioids (86)
  2.6. Pharmacological agent properties and the feedback loop (86)
  2.7. Hypothermia and hypometabolism research and clinical implementation: important considerations (88)
3. Conclusions (89)
Acknowledgements (89)
References (89)

## 1. Introduction

Metabolic homeostasis is key to physical function, justifying its meticulous regulation and powerful governing mechanisms in every living cell. The ability to artificially and reversibly reduce metabolism could provide many advantages for medicine, sports, and aviation. However, despite a growing understanding of our ability to regulate the mechanisms that govern metabolism at the cellular level, translation of metabolic control in cells to a whole organism has remained beyond our reach.

**Figure 1.**
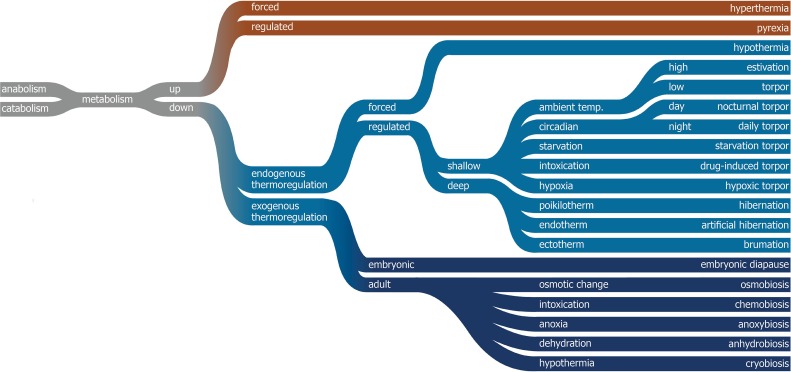
Classification of the different types of hyper- and hypometabolism and the official biological vernacular. Endogenous thermoregulation occurs through the modulation of the thermoneutral zone (Z_tn_) and thermal effectors, whereas exogenous thermoregulation is dependent on the ambient temperature (T_a_) and exogenous triggers but not the Z_tn_.

In nature, reversible states of hypometabolism are a common trait. Members of eight species, including a variety of rodents, carnivores (bears), and primates (lemurs) are known to exhibit a type of hypometabolism [1]. Although biological vernacular varies between many different types of hypome- tabolism (Figure 1), two types can generally be distinguished within animals that have an endogenous thermoregulatory system: a deep and a shallow type of hypometabolism. The difference lies in the depth of the drop in body temperature (T_b_) and the duration. Shallow hypometabolism represents a temporary type, as exhibited during torpor by e.g., the house mouse, whereas deep hypometabolism is a more sustainable type, as is found in e.g., the hibernating ground squirrel [1, 2].

The mechanism(s) responsible for the induction of hypometabolism remain(s) controversial [1]. Unfavorable environmental circumstances appear to be a common denominator in hibernating animals, including seasonal cooling, light deprivation, and prolonged starvation. However, the use of such external triggers to induce artificial hibernation in humans has proven to be of no avail. Irrespective of the external cues, an internal physiological signal, or perhaps a concerted cascade of signals, must initiate, propagate, govern, and sustain hypometabolic signaling *in vivo*. In an attempt to find such a signal, much research has focused on (bio)chemical signaling during hibernation and its induction, leading to the identification of several hypometabolic agents [3-7]. However, despite their discovery and extensive research in various animal models, none of the identified agents appear to induce a hypometabolic state ubiquitously across species, as a result of which not a single hypometabolic agent has made it yet to (pre)clinical application. Currently, the only metabolic control that is clinically employed is forced hypothermia-induced hypometabolism, although this type of hypometabolism fails to achieve the depth found in natural hibernators and comes with challenging limitations.

In an attempt to expand on current insights into hypometabolism, this review addresses the conditions necessary for the induction of hypometabolism and the possible initial (biochemical) triggers. Accordingly, a theorem on the induction of hypometabolism is presented, whereby the relationship between key physiological and environmental factors is provided as a framework to explain the physiological cascade that leads to a sustainable and reversible state of hypometabolism in mammals. Readers should note that the focus of this paper is on mainly the physiological and biochemical conditions required for the induction of hypometabolism. Although neurological signal relay is key to convey, propagate, and sustain a state of hypothermia and hypometabolism, the responsible neurological pathways have only been superficially addressed. These pathways will be elaborated in more detail in a subsequent, separate review.

## 2. A model for hypometabolic induction

### 2.1. Control of metabolism through temperature and substrate availability

**Figure 2.**
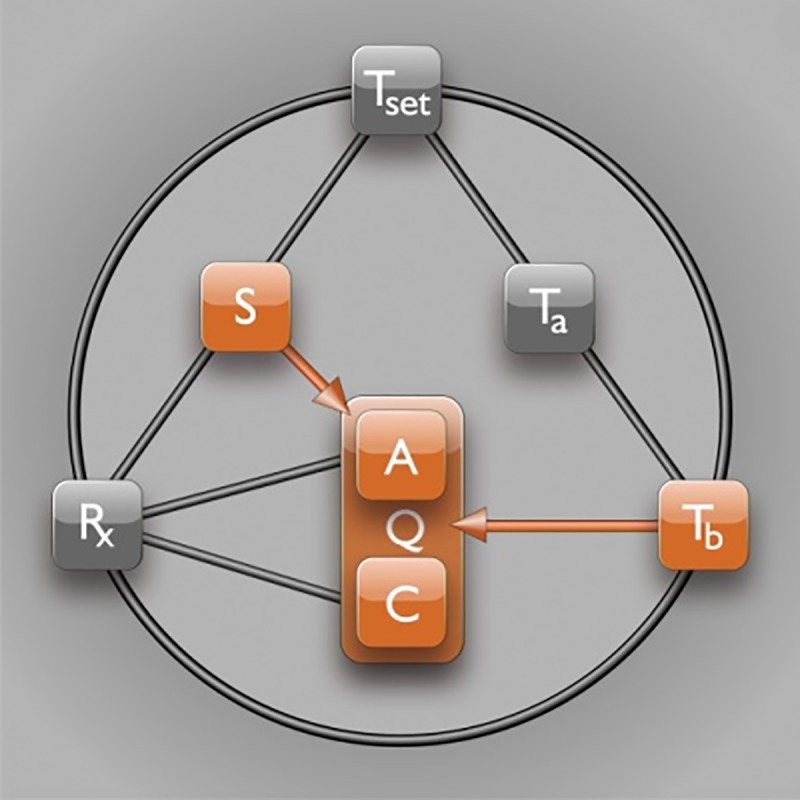
Substrate and temperature effects on metabolism. The S—Q relationship relies on substrate (S) availability to support anabolism (A). The T_b_—Q relationship is dictated by the Arrhenius equation (Equation 1, where *k* is equal to Q in this model), which governs the relation between body temperature (T_b_) and chemical reaction speed (Q). Catabolism (C) is directly affected by T_b_, but not by S.

A pivotal step in the induction of artificial hypometabolism is gaining control over the most important factors that regulate metabolism (Q), namely the availability of substrate (S, i.e., oxygen and glucose) and the rate of adenosine triphosphate (ATP) production (anabolism, A) and consumption (catabolism, C), which are both influenced by the core body temperature (T_b_). Hence, a direct relationship exists between T_b_ and Q (Figure 2) as well as between S and A(Figure 2). The relationship between T_b_ and Q essentially abides by Arrhenius’ law, which states that the chemical (i.e., enzymatic) reaction rate, Q, is reduced as a result of lowering of temperature (Equation 1).

**Equation 1**

Arrhenius equation*:*

**Table:** 

*k*	Rate constant (s^-1^), identical to Q in the proposed model (Figure 11)
A	Prefactor (s^-1^)
E_a_	Activation energy (J·M^-1^), potentially affected by R_x_ in the proposed model (Figure 11)
T	Temperature (K), determined by T_b_ in the proposed model (Figure 11)
R	Universal gas constant (J·K^-1^·M^-1^)

Although the magnitude of Q varies among enzymes, all have in common that Q is temperature-dependent and therefore relies on T_b_. The directly proportional effect of T_b_ on Q is in turn affected by the ambient temperature (T_a_), which impacts T_b_ and hence Q through heat exchange (Figure 2). The (T_a_—)T_b_— Q relationship is widely exploited in the clinical setting, as exemplified by the contrived reduction in patients’ T_b_ through direct or indirect cooling (e.g., reduction in T_a_ by means of breathing cold air, cutaneous cooling, organ perfusion with a cold solution, or intravascular cooling) as a protective strategy in surgery [8, 9], neurology [10], cardiology [11], trauma [12], and intensive care [13]. The protective effects of mild to moderate hypothermia (T_b_ reduction to ~35-32 °C) have been ascribed to lower radical production rates, ameliorated mitochondrial injury/dysfunction, reduced ion pump dysfunction, and cell membrane leakage, amongst others [14]. The majority of these factors is directly related to the rate at which chemical reactions proceed, whereby cytoprotection is conferred by a T_b_-mediated reduction in Q in accordance with the Arrhenius equation (Equation 1).

The generally protective effects of hypothermia notwithstanding, the advantage of clinically forced hypothermia-induced hypometabolism is questionable in some instances. At mild hypothermia (~35 °C), serum concentrations of norepinephrine start to rise in response to hypothermic stress, coagulopathy starts to develop, susceptibility to infections increases, and mortality rates are negatively affected [15-17]. When the T_b_ is lowered further to ~30 °C, severe hypothermia-related complications may occur, including ventilatory and cardiac arrest [14,18]. An even more profound reduction in T_b_ would require mechanical ventilation with extensive monitoring and would considerably increase procedural risks. Accordingly, in the last five decades, the limit of ~30 °C has not been adjusted downward in the clinical setting as much as it has been refined, despite of successful animal experiments with much deeper hypothermia [19-22].

The detrimental effects associated with forced deep hypothermia reflect the limits of the practical implementation of the (T_a_—)T_b_—Q relationship. It is evident that the (T_a_—)T_b_—Q relationship must be differentially regulated in natural hibernators compared to humans. In natural hibernators the T_b_—Q effects may be integratively mediated by endogenous signaling, such as by the release of biochemical agents or by hypoxia (both addressed in detail below). Humans essentially lack such endogenous pathways and do not exhibit hypothermia-related benefits from exogenously administered pharmaceuticals or hypoxia, as a result of which Q cannot be actively adjusted downward by other pathways than through the T_b_—Q relationship. The distinctive responsiveness to hypothermia between humans and natural hibernators may be due to differential neurological and biochemical regulation of T_b_, both of which act via mechanisms related to thermogenesis and heat loss. Thermoregulation and the role of thermogenic and heat loss effectors is therefore addressed in the following section.

### 2.2. Thermoregulation following a shift in the thermoneutral zone

**Figure 3.**
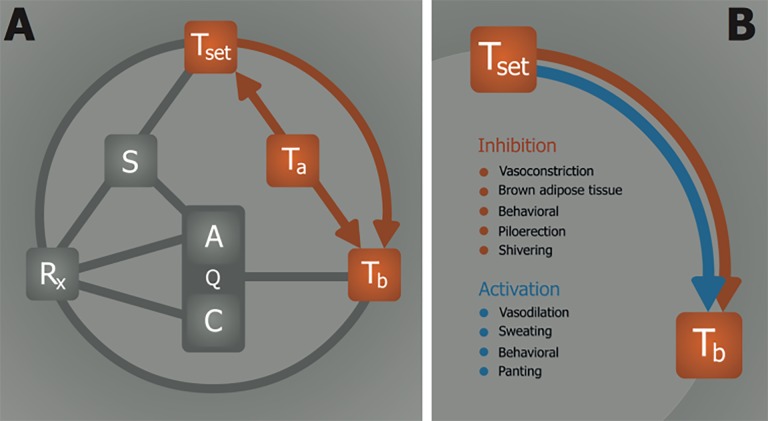
The relationship between the thermoneutral zone and temperature. (A) Overview of thermoregulatory processes. The T_a_—T_b_ relationship represents heat exchange between ambient (T_a_) and body temperature (T_b_). Information on the T_a_ is processed and translated into a thermoneutral zone (Z_tn_) through the T_a_—Z_tn_ relationship, whereby the Z_tn_ maintains T_b_ by regulating thermal effector activity via the Z_tn_—T_b_ relationship. (B) Summary of thermal effectors that are mediated by the Z_tn_—T_b_ relationship. The Z_tn_ activates thermogenic processes (red) when the T_b_ < Z_tn_ and heat loss mechanisms (blue) when the T_b_ > Z_tn_.

The chief role of thermoregulation is maintenance of T_b_ to support an optimal thermodynamic environment for all chemical reactions in the organism, which is around 37 °C in humans. Thermogenic control is believed to rely on several neurological pathways, which includes involvement of the preoptic anterior hypothalamus (POAH). Together, these pathways manage a thermoneutral zone (Z_tn_) which provides a range in which the T_b_ is to maintain itself, and outside of which the T_b_ is to be adjusted towards the Z_tn_ through the use of thermogenic effectors and heat loss effectors (Figure 3).

Although it is difficult to ascertain that a single location among the thermoregulatory pathways can have an effect on the Z_tn_, it is generally accepted that the POAH exerts such an effect in hibernating and non-hibernating animals based on indirect experimental and clinical evidence [23,24]. In humans, incidental but selective destruction of the hypothalamic region is associated with dysfunctional thermoregulation, as evidenced by passive T_b_ declines to as low as 29 °C [25-29]. Moreover, exposure of hypothalamically impaired patients to low T_a_s causes a drop in T_b_, whereas the same conditions induce a rectifying rise in T_b_ in ‘control’ subjects [28,30], attesting to impaired thermoregulatory capacity in hypothalamically afflicted patients. Altogether, these reports provide compelling evidence for a temperature integration site in the hypothalamus through which management of Z_tn_ and T_b_ ensures maintenance of euthermia.

On the basis of these findings it can be concluded that POAH-affected subjects exhibit a sensory defect between Z_tn_— T_a_ (Figure 3A), as a result of which T_b_ will approximate T_a_ in the absence of thermoregulation. Contrastingly, healthy subjects exhibit a reactive effect between Z_tn_—T_b_, whereby T_b_ is sustained in conformity with the Z_tn_ irrespective of the T_a_ via activation of thermogenic effectors (Figure 3B). Accordingly, these data imply that, under normophysiological circumstances, Q is mainly regulated by the Z_tn_ via Z_tn_—T_b_—Q such that the optimal thermodynamic conditions (37 °C) are at all times maintained. POAH-mediated thermoregulation also takes place in rodents that are capable of entering a state of torpor. Corroboratively, selective infarction of the anterior hypothalamus in rats coincides with the inability to regulate T_b_, indicating destruction of pathways that govern the Z_tn_ and abrogation of thermoregulatory function [31].

As opposed to humans, Z_tn_ management in smaller animals (e.g., rodents) may veer from a euthermic regime in some species due to specific changes in environmental conditions. One exemplary condition is hypoxia, which is addressed in section 2.3 to illustrate the relationship between S (oxygen) and Q.

Before moving to the S—Q relationship in the context of hypoxia, however, it is imperative to address hypothermia as a function of an organism’s surface:volume ratio, or the ease with which heat exchange between T_b_—T_a_ can proceed. Small animals have a high surface:volume ratio compared to large animals, which allows for faster heat dissipation and results in subsequent lowering of T_b_ when exposed to cold environments. A high convective efficiency is essential for the induction of hypothermia, and is dependent on the heat loss properties such as the animal’s skin phenotype, breathing pattern, the extent of skin exposure, and the animal’s posture and physical activity [32]. In addition to such effects, small animals require a higher metabolic rate than larger animals to sustain their T_b_ (Kleiber’s law [33]), which causes small animals to become more easily affected by low T_a_s. As a result, it takes considerably less time to lower the T_b_ and coincidentally the Q of a mouse compared to those of a human. This effect is reflected in the strong correlation between the ability and depth of hibernation and surface:volume ratio, which indicates that virtually all hibernating animals are small (i.e., high surface:volume ratio) and that increased body size is associated with a decreased depth of the T_b_ drop during hibernation/torpor [34]. Hence it appears that environmental/biochemical modulation of metabolism is more prevalent in small animals and subject to the effectiveness of T_b_—T_a_ heat exchange.

### 2.3. Hypoxia-induced hypometabolism: aligning anabolism with catabolism

**Figure 4.**
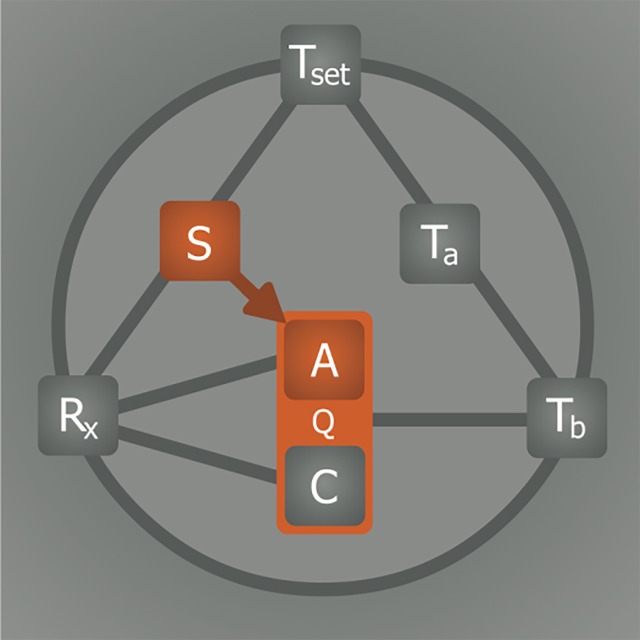
Effects of hypoxia on metabolism. Metabolism (Q) is controlled by substrate (S) availability. Lowering of oxygen availability (hypoxia) directly inhibits anabolism (A, i.e., ATP production) but not catabolism (C, i.e., ATP consumption). Consequently, the metabolic tolerance of hypoxia is limited by the extent to which C can be sustained in the absence of A.

The relationship between S and Q is to an extent regulated by the intracellular oxygen tension insofar as oxygen constitutes a vital S for Q (Figure 4). During hypoxia, Q is impaired because of insufficient oxygen availability for oxidative phosphorylation, resulting in reduced cytochrome c oxidase function [35] and cessation of ATP production (A). Under hypoxic but normothermic conditions, the ATP consumption rate (C) can be suppressed to match the ATP production rate, but only to a limited extent and for a short time [36]. In order to survive during a long period of normothermic hypoxia, an organism’s metabolism must remain active to fuel metabolically vital processes, which include protein synthesis (25-30% of total ATP consumption), ion homeostasis (23-36%), gluconeogenesis (7-10%), and ureagenesis (3%) [37]. The limited production yet active consumption of ATP during normothermic hypoxia therefore forces the organism to initially switch to anaerobic respiration (Pasteur effect) - a switch that imposes limits on the maximally tolerable duration of hypoxia/anoxia due to inefficient ATP yields from glycolysis and the production of toxic metabolites such as lactic acid.

**Figure 5.**
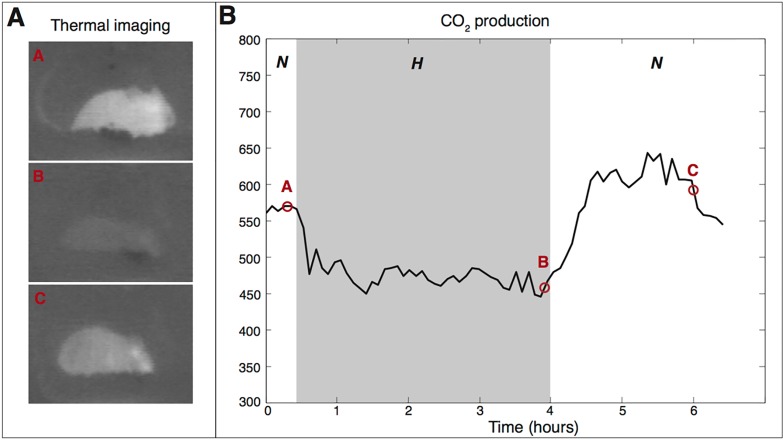
Induction of hypometabolism by hypoxia in mice. An experiment was conducted that exemplifies the reduction in temperature (measured with a thermal camera) and metabolism (measured by exhaled CO2 levels) following exposure of mice (*Mus musculus*) to hypoxia. The mouse was placed in an air-tight container with an inlet coupled to a gas cylinder containing either an O_2_:N_2_ mixture of 21% O_2_ (to induce normoxic conditions, *N*) or an O_2_:N_2_ mixture of 5% O_2_ (to induce hypoxic conditions, *H*). The container was purged with the normoxic or hypoxic gas mixture at a flow rate of 1 L/min. The container also had a gas outlet that was coupled to a CO_2_ sensor (model 77535 CO_2_ meter, AZ Instrument, Taichung City, Taiwan). After a 30-min stabilization period under normoxic conditions, hypoxia was induced for 3.5 h, after which the container was changed back to normoxic conditions and the mouse was allowed to recover for an additional 3 h. The ambient temperature (T_a_) was maintained at 23.4 ±0.3 °C. During the experiment the mouse was imaged with a thermal camera (Inframetrics, Kent, UK), whereby dark pixels indicate low temperatures and light pixels indicate high temperatures. The frame designations correspond to the lettering in the CO_2_ production chart to indicate the time point and phase at which the images were acquired. Upon induction of a hypoxic environment, the body temperature (T_b_) of the mouse dropped (B), as evidenced by the decreased T_b_-T_a_ contrast between 0.5 h and 4 h. Following restoration of normoxic conditions (C), the animal’s T_b_ gradually returned to baseline levels. The right panel shows the CO_2_ profile during normoxia and hypoxia, whereby the hypoxic phase is clearly associated with reduced levels of exhaled CO_2_, which constitutes a hallmark of hypometabolism.

**Figure 6.**
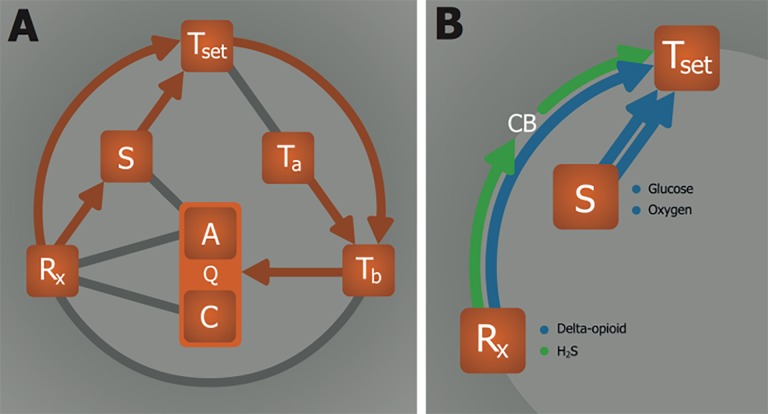
Hypoxia-induced hypometabolism via anapyrexic signaling. (A) The onset of hypoxia, i.e., low substrate (S = oxygen) levels, is proposed to modulate the thermoneutral zone (Z_tn_) downward via a so-called hypoxic link. The lowering of the Z_tn_ inhibits thermogenesis and activates heat loss mechanisms through the Z_tn_—T_b_ relationship, allowing heat exchange between body temperature (T_b_) and ambient temperature (T_a_) to occur. The consequent reduction in T_b_ slows down both anabolic (A) and catabolic (C) metabolism (Q) as described in Figure 2. (B) Pathways leading to anapyrexia via the R_x_—Z_tn_ and S—Z_tn_ relationships. Although this relationship is exemplified for hypoxic conditions, where S comprises oxygen, it may also apply to conditions where another S is reduced, such as glucose during periods of starvation. Prolonged hypoglycemia is known to also induce hypometabolism, as addressed in section 2.5.4. A direct anapyrexic pathway is suggested for R_x_—Z_tn_, where a neuroactive agent such as delta-opioids directly lowers the Z_tn_ (section 2.5.5). Alternatively, an R_x_ such as H_2_S can also affect the Z_tn_ without affecting S availability by inducing hypoxic signaling through oxygen sensors such as carotid bodies (section 2.5.1).

In several non-hibernating species, exposure to a prolonged period of hypoxia concurs with regulated hypothermia and hypometabolism, suggesting that hypoxia-mediated thermogenic and metabolic suppression constitutes a protective/ coping mechanism for such life-threatening conditions [38-41]. This hypoxic stress response, illustrated in Figure 5, is in fact an effective survival mechanism in that the catabolic rate is realigned with the limited anabolic rate caused by hypoxia, which is in part achieved by the lowering of T_b_ through the inhibition of thermogenesis and activation of heat loss mechanisms (explained in section 2.4). In that respect, the hypoxic stress response essentially embodies a pre-programmed manifestation of Arrhenius’ law. During this process, the Z_tn_ must either shift downward or be biochemically inhibited in order to resolve the incongruity between the hypothermic T_b_ and the euthermically ranged Z_tn_. A reduction in T_b_ (and consequently Q) resulting from a downward adjustment of the Z_tn_ is referred to as anapyrexia (Figure 6A), as opposed to pyrexia, which comprises an elevated T_b_ as result of an elevated Z_tn_ (i.e., fever). How anapyrexia is mediated under hypoxic conditions in animals is elusive. The effect of anapyrexia on thermoregulatory effectors, on the other hand, is not and provides useful information on the hypoxia-anapyrexia signaling axis.

### 2.4. The effect of hypoxia-induced anapyrexia on thermoregulatory effectors

Hypoxia-induced anapyrexia is found in a large number of species, including mice [39,42], hamsters [43], rats [38,44], pigeons [45,46], dogs [42,47], primates [41], and man [42], and manifests itself when the organism is concurrently exposed to low T_a_. A low T_a_ appears to be a prerequisite for an anapyrexic response to hypoxia, as an anapyrexic response during hypoxic euthermic conditions is absent. The main question, however, is how hypoxic signaling decreases the Z_tn_ to facilitate hypothermia.

The answer may entail an effect of hypoxia on thermogenic effectors (Figure 6A), such as BAT and shivering (Figure 3B). The inhibitory effects of hypoxia on the intensity of cold-induced BAT activity include a lower afferent blood flow to BAT [48], reduced sympathetic nerve activity [49], desensitized response to norepinephrine (a potent BAT activating agent [50,51]) [52], and can eventually lead to a reduction in BAT mass during prolonged exposure to hypoxia [53,54]. In addition, hypoxia results in the inhibition of shivering upon exposure to low T_a_ compared to normoxic controls in mice, dogs, and man [42]. Moreover, some species further reduce their T_b_ through changes in behavioral patterns, such as disengagement from cold-induced huddling [55] or exhibiting an explicit preference for cooler environmental temperatures [56].

Considering these effects, it can be hypothesized that hypoxia either acts directly on BAT and muscle tissue (shivering) or indirectly inhibits these thermogenic effectors via central regulation, the Z_tn_. The latter is a more likely mechanism of action since anapyrexic signaling controls both BAT and shivering in order to facilitate hypothermia. Poor blood oxygenation, a result of exposure to hypoxia, is relayed to the brain via the carotid bodies, which is described by a R_x_—Z_tn_ relationship (Figure 6B). CBs are oxygen sensing bodies located alongside the carotid artery that contain oxygen-sensitive chemoreceptors through which they provide essential neuronal feedback on the arterial partial oxygen pressure [57]. Excitation of the CBs by reduced oxygen levels during hypoxia possibly induces lowering of the Z_tn_ to activate heat loss effectors(Figure 3B) so as to facilitate the induction of hypothermia with the sole purpose of aligning ATP consumption rates with ATP production rates as part of the survival response to stress conditions (section 2.3). Naturally, this response prevails in species that have a sufficiently high T_a_—T_b_ convective efficiency to allow rapid manifestation of hypothermia and corollary reduction in Q (section 2.2), given that sustenance of life by anaerobic metabolism is time-limited. CBs are therefore an important instrumental component of the ‘hypoxic link’ in smaller species.

The existence of a ‘hypoxic link’ to the Z_tn_ (Figure 6) has been suggested [58] but lacks direct evidence other than the previously mentioned changes in thermal effectors. This link implies that, under cold but normoxic conditions, the Z_tn_ enforces an array of physiological tools that coordinate a thermogenic response, such as shivering, activation of BAT, vasoconstriction, and piloerection (Figure 3B). Under hypoxic conditions, however, these thermogenic responses are dampened or even absent and coincide with activation of heat loss effectors such as vasodilation, sweating, and panting (Figure 3B) [58]. Direct measurement of changes in the Z_tn_ range would be useful in substantiating the ‘hypoxic link.’ Unfortunately, due to incomplete knowledge of Z_tn_ functionality and technical difficulties related to reaching the neural pathways involved, it is currently very difficult to directly measure the range of the Z_tn_ or changes therein.

In summary, hypoxia (low S levels) leads to hypometabolism potentially by signaling anapyrexia through CBs, thereby allowing the body to cool via the S—Z_tn_—(T_a_—)T_b_—Q relationship. The hypoxia-induced anapyrexic component provides an advantage over the T_b_—Q relationship in that the anapyrexia allows the T_b_ to drop below normothermia, preventing the stress response that would otherwise be needed to keep the body normothermic. Nevertheless, it is unlikely that these pathways constitute all necessary conditions for the induction of natural hibernation. A closer look at (bio)chemical agents (R_x_) that have the ability to induce or mimic ‘anapyrexiadriven hibernation’ present additional pathways, namely through their effect on S availability, the relay of S availability to the Z_tn_, and through direct effect on Z_tn_.

### 2.5. Pharmacological agents and induction of artificial hypometabolism

**Figure 7.**
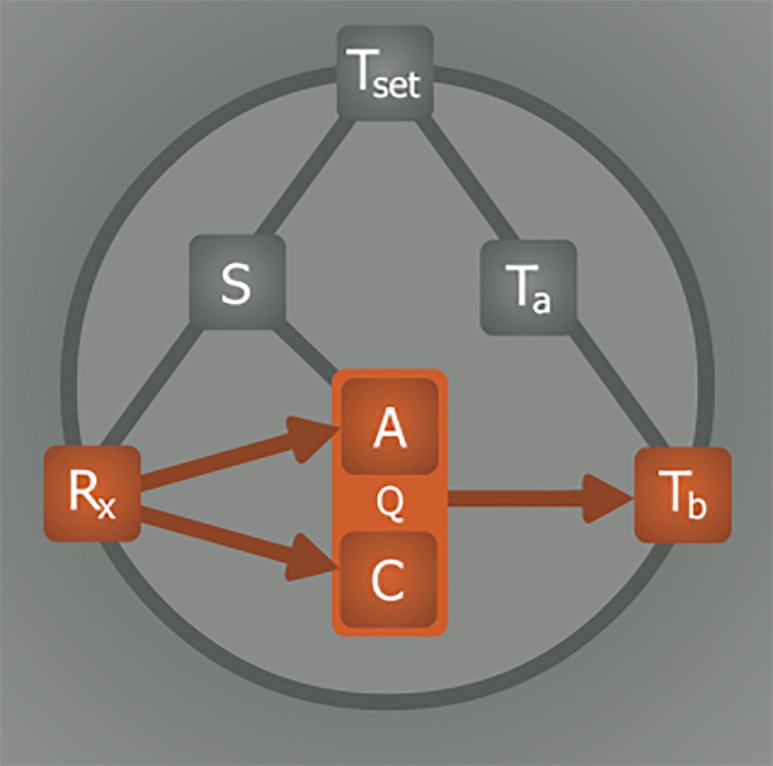
The effect of hibernation induction trigger on metabolic activity. It has been suggested that metabolism (Q) may be directly inhibited by pharmacological agents (R_x_) such as hibernation induction trigger (HIT) via inhibition of anabolism (A) and/or catabolism (C). It should be underscored that, based on the information presented in sections 2.2 and 2.4, this pathway is unlikely to occur in the absence of hypothermic signaling via Z_tn_ downmodulation.

Induction of hypometabolism in natural hibernators normally occurs in response to environmental triggers such as low T_a_ and light and/or food deprivation. The internal (bio)chemical trigger responsible for the subsequent propagation of this signal is key to understanding the hibernation process. It has been suggested that a yet to be characterized endogenous molecular compound, referred to as hibernation induction trigger (HIT), is responsible for inducing hibernation *in vivo* [59,60] via the R_x_—Q(—T_b_) relationship, whereby the drop in T_b_ is arguably a consequence of the reduced Q by the HIT (Figure 7). It should be noted that this mechanism would differ fundamentally from anapyrexic signaling, which requires Z_tn_ downmodulation and subsequent T_a_—T_b_ equalization as a precursor event for hypometabolic induction (Figure 6). In an effort to identify the HIT, different endogenous compounds have been investigated that have potential to account for or mimic the effect of the HIT, including H_2_S [3], 5’-adenosine monophosphate (5’-AMP) [61], thyronamines (TAMs) [6], 2’-deoxyglucose (2-DG) [4], and delta-opioids (DOPs) [5, 59].

**Figure 8.**
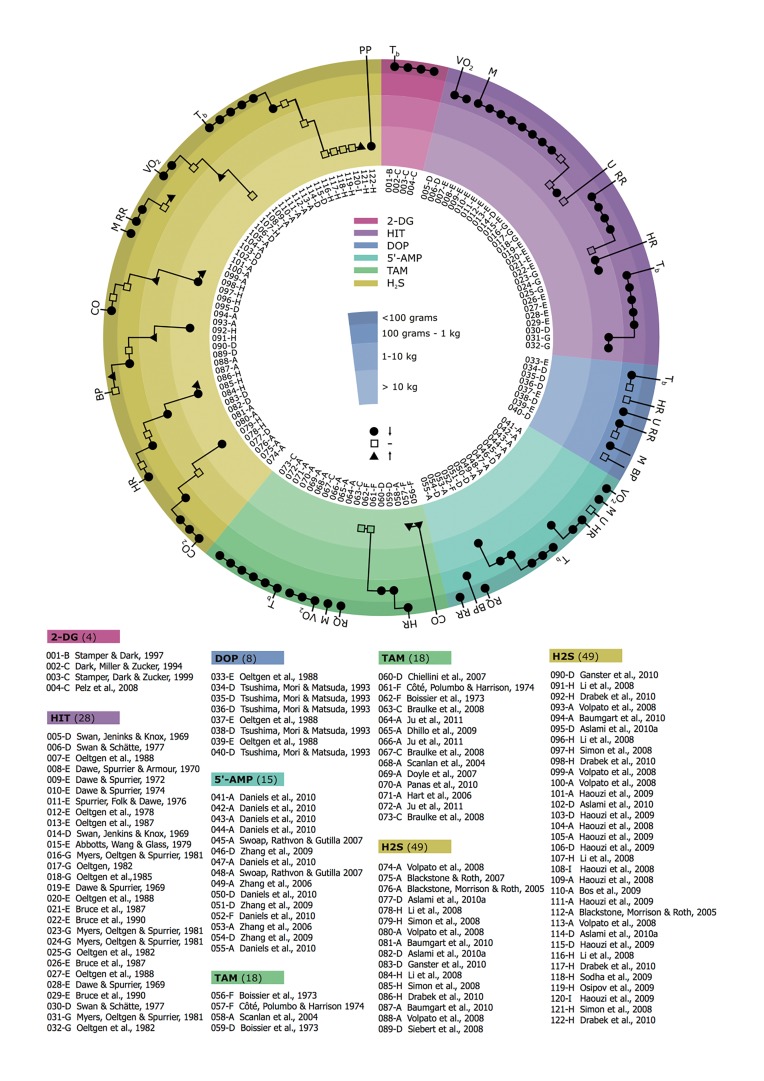
Physiological effects of hibernation inducing agents. Overview of physiological effects in response to 2-deoxyglucose (2-DG), 5’-adenosine monophosphate (5’-AMP), hydrogen sulfide (H_2_S), hibernation induction trigger (HIT), delta-opioid (DOP), and thyronamine (TAM). Each effect is represented by a black dot (lowering of value), transparent square (equal value), or black triangle (rise of value). The effects are stratified according to the size of the animal, from outer ring to inner ring these are: < 0.1 kg (e.g., mouse), 0.1-1 kg (e.g., rat), 1-10 kg (e.g., macaque) and > 10 kg (e.g., pig). The inner white ring indicates the respective reference (number) and species (letter). Animals: A, house mouse (Mus musculus, Linnaeus), B, deer mouse (Peromyscus maniculatus, Wagner); C, djungarian hamster (Phodopus sungorus, Pallas); D, common rat (Rattus novergicus, Berkenhout); E, thirteen-lines ground squirrel (Spermophilus tridecemlineatus, Mitchill); F, domestic dog (Canis lupus familiaris, Linnaeus); G, rhesus macaque (Macaca mulatta, Zimmermann) or southern pig-tailed macaque (Macaca nemestrina, Linnaeus); H, domestic pig (Sus scrofa domesticus, Erxleben); I, sheep (Ovis aries, Linnaeus). The physiological parameters include: T_b_, core body temperature; VO_2_, oxygen consumption; M, motion; RR, respiratory rate; HR, heart rate; U, urine production; BP, blood pressure; RQ, respiratory quotient; CO, cardiac output; CO_2_, carbon dioxide production; PP, pulmonary pressure. All parameters are expected to be reduced during hypometabolism.

Although induction of a hypometabolic state is a shared trait of these agents, each is associated with a different pattern of physiological effects. The physiological effects related to hibernation, as summarized in Figure 8, are primarily found in small animals (Figure 8, outer ring) and not so much in large animals (Figure 8, inner ring). The high incidence of hypometabolic effects in small animals suggests that part of the R_x_ mechanism may rely on anapyrexia according to the R_x_— (S)—Z_tn_—T_b_—Q relationship described in Figure 6, and underscores the importance of the surface:volume ratio (section 2.2). Consequently, the currently identified hypometabolism-inducing agents are addressed in relation to their direct anapyrexic properties (R_x_—Z_tn_), their indirect anapyrexic properties (R_x_—S—Z_tn_), or their substrate affecting properties (S—A).

#### 2.5.1. Hydrogen sulfide

Exposure to H_2_S consistently produces a hypometabolic state in small animals such as mice and rats (Figure 8, outer ring) [3,62-66]. However, the use of H_2_S in larger animals such as pigs [67-70], sheep [65], and heavy rats [71] has failed to induce a hypometabolic response (Figure 8, inner ring). The current mechanistic paradigm of the hypometabolic effect of H_2_S is based on its high membrane permeability and direct inhibitory effect on cytochrome c oxidase in the electron transport chain (i.e., through the R_x_—A relationship) [72,73]. However, increasing evidence indicates the hypometabolic effects are the result of hypoxic signaling. For example, endogenously produced H_2_S is necessary for CBs to signal hypoxia [74]. Exogenously applied H_2_S, via a soluble NaSH precursor, can mimic the production of this hypoxic signal *in vitro* [75], suggesting the utilization of the ‘hypoxic link’ (R_x_—Z_tn_) by H_2_S (Figure 6B). This is supported by the observed dichotomy between H_2_S-induced effects in small versus large species, where H_2_S produces hypometabolic effects in small species but not in larger species (Figure 8). The R_x_—Z_tn_—T_b_—Q relationship is dependent on a high T_a_—T_b_ convective efficiency; a property that prevails strictly in small species due to their high surface:volume ratio (section 2.2).

#### 2.5.2. Adenosine monophosphate

Intraperitoneal injections of 5’-AMP have been shown to induce an artificial hypometabolic state in mice and rats as evidenced by a profound drop in T_b_ (Figure 8) [61,76-78]. The putative contention is that intraperitoneal administration of high 5’-AMP concentrations (e.g., 500 mg/kg) lead to extensive 5’-AMP uptake by erythrocytes [79], after which the high intracellular levels of 5’-AMP drive the adenylate equilibrium (ATP + AMP ↔ 2 ADP) towards production of ADP, thereby depleting erythrocyte ATP levels [76,77]. As a result, erythrocyte 2,3-disphosphoglycerate is upregulated, limiting the binding of oxygen to hemoglobin’s oxygen binding sites (referred to as oxygen affinity hypoxia) [76]. In addition to the already impaired oxygen transport, the severe cardiovascular depression following 5’-AMP administration has the potential to further exacerbate this hypoxic state (referred to as circulatory hypoxia) [78]. Although there is no conclusive evidence that these types of hypoxia have the ability to induce an anapyrexic state, the generally pervasive hypoxic state likely uses the S—Z_tn_—T_b_(—T_a_)—Q relationship (Figure 6A) to induce hypometabolism through CB signaling.

More recent studies have implicated a direct 5’-AMP signal transduction route to the central nervous system in seasonal hibernators, culminating in the induction of torpor [80-83]. 5’-AMP signaling occurs via the A_1_ adenosine receptor (A_1_AR), which is ubiquitously distributed throughout all tissues, but not the A_2a_AR or A_3_AR receptors. In the brain, A_1_AR signaling leads to deceleration of metabolic rate and induction of a torpor state in arctic ground squirrels [84]. In this species, intracerebroventricular administration of the A_1_AR antagonist cyclopentyltheophylline reversed spontaneous entrance into torpor during hibernation season, whereas administration of the A_1_AR agonist N(6)-cyclohexyladenosine induced torpor [82]. Later studies confirmed the manifestation of 5’-AMP-driven torpor in non-hibernators, including the mouse [80] and rat [83], suggesting that the hypothermia- and hypometabolism-inducing potential of 5’-AMP is pleiotropically applicable across species. Although the signaling is of more direct neurochemical nature compared to the hypoxic signaling, the S—Z_tn_—T_b_(—T_a_)—Q relationship (Figure 6A) also holds for the 5’-AMP-A_1_AR signaling axis.

As indicated previously, the depth of the hypometabolic response is dependent on the T_b_—T_a_ convective efficiency (section 2.2). This is further supported by the extent of the drop in T_b_ that is observed in 5’-AMP-treated mice, which is proportional to the difference between T_b_ and T_a_ (i.e., lower T_a_s induce a greater drop in T_b_), demonstrating the T_b_—T_a_ dependency [76].

#### 2.5.3. Thyronamines

TAMs are a thyroid hormone-derived group of compounds of which currently nine structural analogues have been identified [6]. Contrary to the structurally similar metabolism- enhancing thyronines (T_3_ and T_4_), exposure to TAM analogues triggers a transient T_b_ depression in small animals, epitomizing the induction of a hypometabolic phase (Figure 8) [6,85-88]. Although earlier studies in a canine model presented contradictory evidence with respect to metabolic effects compared to later studies (Figure 8, cardiac output), it is likely that this was a result of differences in synthesis methods and compound purity [87,89]. Nevertheless, the metabolic effects of TAMs remain obscure, regardless of the synthesis method.

Both *in vivo* and *ex vivo* studies have found the physiological effects of TAMs to be mainly cardiogenic in nature, producing severe hemodynamic depression, bradycardia, hypotension, and reduced cardiac output [6,85,90]. These effects result in reduced oxygen levels (affecting the R_x_—S—Z_tn_ relationship, Figure 6) by lowering the extent of blood oxygenation in accordance with Fick’s principle, which describes an inverse relationship between cardiac output and oxygenation (circulatory hypoxia) [91, 92]. Although the effect of circulatory hypoxia on the Z_tn_ has not been demonstrated, the presence of hypoxia in the broader sense may support the implication of the S—Z_tn_—T_b_(— T_a_)—Q relationship (Figure 6A) as a basis of the observed hypometabolism in smaller animals.

Given the magnitude of the hemodynamic collapse in small species, TAMs may additionally render the animal motionless (Figure 8, motion), which would facilitate a greater rate of thermal convection (T_b_—T_a_) and therefore accelerate the consequent reduction in T_b_ and Q (Figure 2).

#### 2.5.4. Deoxyglucose

In addition to oxygen, S can also comprise glucose, as evidenced by the induction of a hypometabolic state upon glucose deprivation in hamsters and various types of mice [4, 93-95]. Shortage of food, and with that the ability to use glucose as energy substrate, has the potential to induce hypometabolism or alter its depth [94-96]. This principle has been demonstrated in hamsters using 2-DG, a glucose analogue that cannot undergo glycolysis as a result of the 2-hydroxyl group, which causes the animals to enter a hypometabolic state [4,93]. The hypometabolic response to 2-DG, measured by the drop in T_b_, is reflective of the S—A relationship (Figure 4). Currently there is no evidence that supports the S—Z_tn_ pathway for 2-DG.

#### 2.5.5. Delta-opioids

Isolation and characterization of the HIT found in serum of hibernating woodchucks and squirrels revealed a resemblance to D-Ala-D-Leu-5-enkephalin, a non-specific DOP receptor agonist [5,97]. Consistent with this characterization, DOPs induce a hypometabolic state of similar depth as the HIT, whereas mu- or kappa-opioid receptor agonists show no effect [97,98]. The involvement of opioid receptors is further substantiated by the inability of DOP to induce hypometabolism upon exposure to naloxon, a non-specific opioid receptor antagonist [99-102]. These findings raise the question whether DOPs make use of a direct R_x_—Q relationship or act via the R_x_—Z_tn_ (Figure 6). Growing evidence suggests the latter, as delta-opioids appear to be directly involved in hypoxic signaling. In mice, exposure to hypoxia has been suggested to decrease Z_tn_ via delta-1-opioid receptor agonism [103-106]. Other studies have suggested that the delta-2 opioid receptor rather than a delta-1 opioid receptor is responsible for the effects on the Z_tn_ [107-109], supported by the limited presence of delta-1-opioid receptors in the hypothalamic region [110,111]. However, in both cases the preferred pathway to Z_tn_ modulation appears to involve a direct R_x_—Z_tn_ relationship.

### 2.6. Pharmacological agent properties and the feedback loop

**Figure 9.**
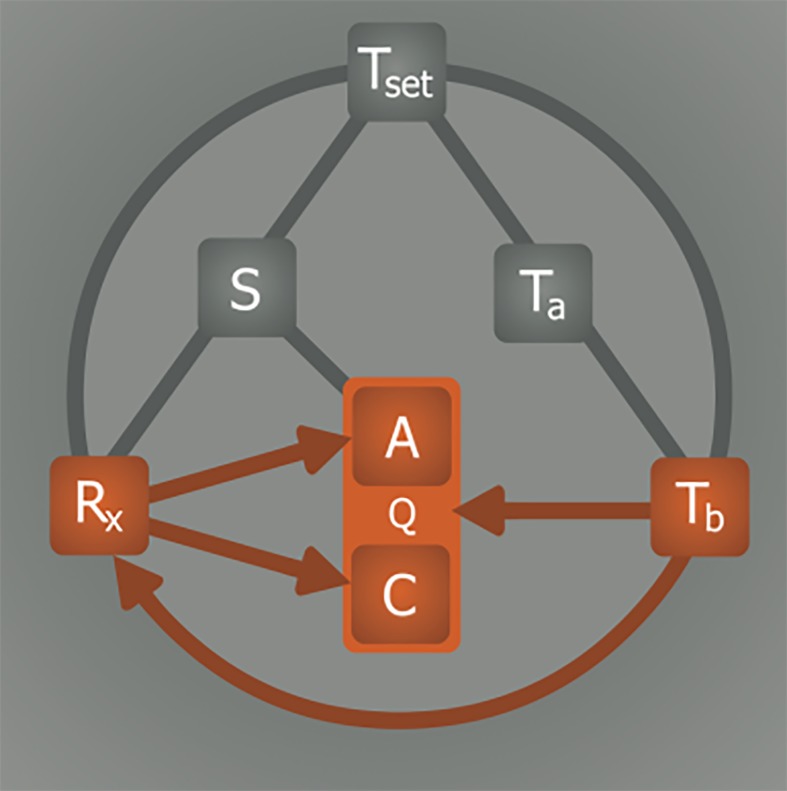
Signal amplifying feedback loop during hypometabolic induction. Theoretically, lowering of the body temperature (T_b_) could be part of a feedback system that triggers the release of a metabolism inhibiting agent (R_x_) capable of further lowering metabolism (Q) via direct inibition of anabolism (A) and/or catabolism (C). This process is embodied by the T_b_—R_x_— Q relationship.

As demonstrated by the different mechanisms discussed in the previous section, R_x_ can affect Q via three potential pathways, namely via R_x_—Q(—T_b_) (Figure 7, suggested for HIT), via R_x_—S—Z_tn_—T_b_—Q (Figure 6, suggested for H_2_S, 5’-AMP, TAM, and 2-DG), and via R_x_—Z_tn_—T_b_—Q (suggested for DOP). However, irrespective of the hypometabolic pathway, it is unlikely that the systemic release of a single R_x_ accounts for the hypothermic/hypometabolic induction process. Instead, as is the case in many biochemical pathways, it is more probable that the induction of hypometabolism is governed by a signal amplifying feedback loop (Figure 9).

With respect to the model, the ideal properties of an R_x_ regarding its regulatory function of the feedback loop encompass 1) endogenous production and/or release during induction of hibernation, 2) inhibitory effects on both A and C, 3) downregulatory effect on Z_tn_, 4) equal distribution throughout the body, and 5) availability of agonists and antagonists to accelerate and abrogate R_x_-mediated signaling, respectively. Although currently there is no sound evidence for the existence of an R_x_ feedback loop, such a mechanism is theoretically necessary to propagate a hypometabolic signal *in vivo*. Consequently, a mechanistic framework for such a feedback loop will be elaborated for magnesium (Mg^2+^), which appears to play an important role in hibernation across different species.

**Figure 10.**
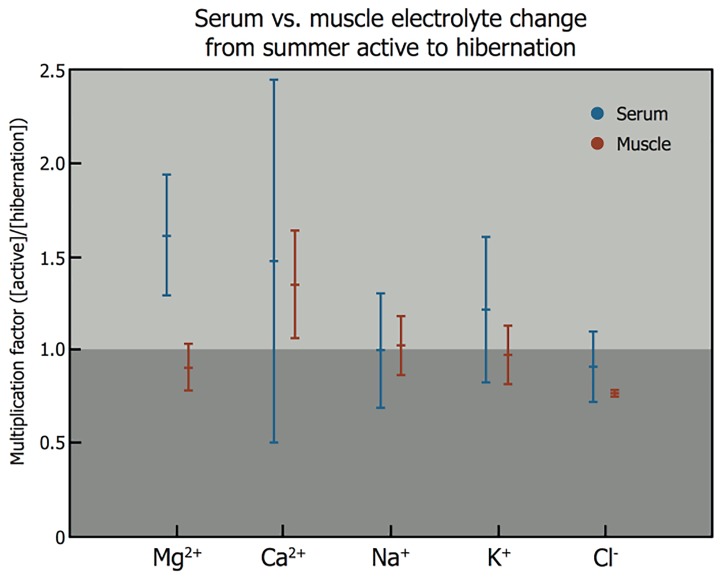
Electrolyte changes during induction of natural hibernation. Analysis of blood levels of magnesium (Mg^2+^, serum *n* = 23, muscle *n* = 9), calcium (Ca^2+^, serum *n* = 19, muscle *n* = 5), sodium (Na^+^, serum *n* = 16, muscle *n* = 6), potassium (K^+^, serum *n* = 25, muscle *n* = 9), and chloride (Cl^−^, serum *n* = 9, muscle *n* = 3) from summer active state to hibernation (< 1, reduction upon induction into hibernation; 1, no change; > 1, increase in electrolyte concentration). Black bars correspond to serum electrolyte levels, grey bars indicate electrolyte levels in muscle tissue. Animals included in this figure are: European hedgehog (*Erinaceus europaeus*, Linnaeus) [114-116], long-eared hedgehog (*Hemiechinus auritus*, Gmelin) [117], golden hamster (*Mesocricetus auratus*, Waterhouse) [118-122], common box turtle (*Terrapene carolina*, Linnaeus) [123], pond slider (*Trachemys scripta*, Thunberg) [123], painted turtle (*Chrysemys picta*, Schneider) [124-127], European ground squirrel (*Spermophilus citellus*, Linneaus) [128], thirteen-lined ground squirrel (*Spermophilus tridecemlineatus*, Mitchill) [121, 122, 129], groundhog (*Marmota monax*, Linneaus) [130, 131], yellow-bellied marmot (*Marmota flaviventris*, Audubon & Backman) [132], Asian common toad (*Duttaphrynus melanostictus*, Schneider) [133], little brown bat (*Myotis lucifugus*, LeConte) [122, 134, 135], big brown bat (*Eptesicus fuscus*, Palisot de Beauvois) [122;134], American black bear (*Ursus americanus*, Pallas) [122], common musk turtle (*Sternotherus odoratus*, Latreille) [136], desert monitor (*Varanus griseus*, Daudin) [137]. Statistical analysis was performed in MatLab R2011a. Intragroup analysis of serum versus muscle electrolyte levels (Mann-Whitney U test: p-value): Mg^2+^, p < 0.001; Ca^2+^, p = 0.395; Na^+^, p = 0.299; K^+^, p = 0.067; Cl^−^, p = 0.315. Intergroup analysis of serum electrolyte levels, indicating statistical differences (Kruskal-Wallis test): Mg^2+^ versus Ca^2+^, Na^+^, K^+^, and Cl^−^, (p < 0.05).

Induction of hibernation coincides with a change in serum Mg^2+^ concentration (Figure 10). Serum Mg^2+^ levels increase by an average of 1.6-fold upon induction of hibernation in different species compared to their summer active state, which is a considerably higher increase than observed for other electrolytes. The release of Mg^2+^ into the circulation occurs from storage pools that have formed prior to induction of hibernation in cells that comprise muscle (Figure 10) and skin tissue [112, 113]. The translocation of Mg^2+^ from tissue to blood and subsequent systemic distribution is in conformity with the first R_x_ property, i.e., the release of an endogenous agent during the induction of hibernation.

In regard to the second property, Mg^2+^ exerts an inhibitory effect on Q, affecting both A and C. Mg^2+^ acts as a necessary co-factor in over 300 enzymatic reactions[138]. When the Mg^2+^ concentration exceeds the saturating concentration required for occupying all substrate binding sites, Mg^2+^ becomes an inhibitor of enzymatic activity [138]. The inhibitory properties of Mg^2+^ are not limited to the inhibition of A, such as reduction of state III respiration (ADP-stimulated respiration) upon exposure to Mg^2+^ [139], but also include inhibition of C, such as reduction of Na/K-ATPase activity [140]. In addition, Mg^2+^ inhibits ion channels, such as the NMDA receptor ion channel and voltage gated ion channels [141-143]. Although inhibition of A and C are essential in sustaining a prolonged state of hypometabolism, as occurs during hibernation, this R_x_ property has been largely ignored in reports on conventional hibernation-inducing R_x_ agents (i.e., H_2_S, 5’-AMP, TAM, 2-DG, and DOP).

The third property of an R_x_ is its downward adjusting effect on the Z_tn_. In case of Mg^2+^ there appears to be conflicting evidence; intracerebroventricular perfusion with a solution containing a supraphysiological concentration of Mg^2+^ does not result in a hypothermic response in hamsters [144], rats [145], cats [146-148], and primates [149, 150] but does result in a hypothermic response in pigeons [151], dogs [152], and sheep [153]. However, with this perfusion approach it cannot be guaranteed that intracerebroventricular perfusion solely affected the neural pathways involved in thermoregulation. A more accurate assessment can be made on the basis of the thermal effectors, in which case Mg^2+^ exerted an inhibitory effect on shivering in cold-exposed dogs [154], reduced postanesthetic shivering in patients [155-157], lowered the coldinduced shivering threshold in healthy human subjects [158], and promoted heat loss effectors in rats [159]. Essentially, these reports constitute indirect evidence for the Z_tn_ lowering property of Mg^2+^, which is manifested through the activation of heat loss mechanisms and inhibition of thermogenic mechanisms (R_x_— Z_tn_—T_b_, Figure 6).

Fourth, an R_x_ must distribute throughout the entire body. Although self-evident, this property is often omitted in common theories on the induction of hibernation by pharmacological agents. Heterogeneously distributed receptors of an R_x_ deter widespread propagation of hypometabolic signaling, and instead support hypometabolic signaling through an interposed effect such as the lowering of the Z_tn_ (e.g., DOP) or the availability of S (e.g., TAM). The systemic distribution of Mg^2+^ is unclear, but is expected to be ubiquitous given the role of this cation in many enzymatic reactions, including in the hexokinase-mediated conversion of glucose to glucose 6-phosphate, which occurs in every somatic cell type as part of sugar metabolism.

Finally, it is important that an R_x_ is sensitive to stimulation and inhibition for the induction and abrogation of hypometabolic signaling, respectively. The natural factors that trigger hibernation include lowering of T_a_ and dietary change, both of which are able to increase plasma Mg^2+^ via cold-stimulated muscular and dermal release of Mg^2+^ that is stored during the pre-starvation diet period [160]. The subsequent rise in plasma Mg^2+^ can promote inhibition of thermogenic activity and activation of heat loss mechanisms by lowering of the Z_tn_, potentiating heat exchange (T_a_—T_b_). In addition, direct effects on shivering via Mg^2+^-mediated inhibition of neuromuscular transmission facilitates lowering of T_b_ [161]. The consequent cooling releases more Mg^2+^ stored in muscles and skin, further adding to the increase in serum Mg^2+^ through a positive feedback loop. As discussed in section 2.2, a feedback loop of this type would be most efficient in small animals due to their high body surface:size ratio and result in a less profound T_b_ drop in larger animals.

### 2.7. Hypothermia and hypometabolism research and clinical implementation: important considerations

**Figure 11.**
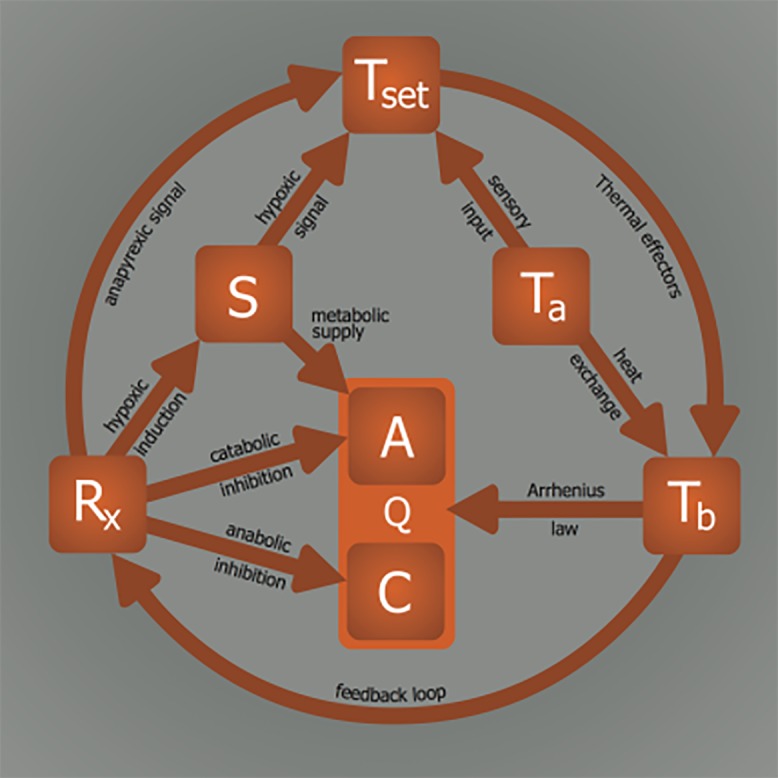
Model for induction of (artificial) hypometabolism. Depicted parameters: Q, overall metabolism defined as chemical reaction speed (i.e., similar to *k* in Equation 1); C, catabolism; A, anabolism; T_b_, core body temperature; T_a_, ambient temperature; Z_tn_, thermoneutral zone; S, substrate; R_x_, (bio)chemical agent able to induce hypometabolism. The relationships: T_b_—Q, Arrhenius law; T_a_—T_b_, heat exchange; Z_tn_—T_b_, thermogenesis and heat loss mechanisms; T_a_—Z_tn_, sensory input; S—Z_tn_, hypoxic link; R_x_— S, hypoxia/hypoglycemia induction; R_x_—C, catabolic modulation; R_x_—A, anabolic modulation; R_x_—Z_tn_, anapyrexic signal; S—A, metabolic substrate supply; T_b_—R_x_, positive/negative feedback loop.

The complete model on the induction of hibernation, presented in Figure 11, is in part hypothetical and requires additional research to validate every relationship. Given the supportive experimental evidence discussed in the previous sections, the model provides a starting framework for interpreting observations made in future *in vivo* experiments concerning hypothermia and hypometabolism, particularly in the context of integrative physiology. There are several important considerations regarding this type of research that must be accounted for, especially when data are interpreted on the basis of the model.

As implied in sections 2.1 and 2.2, prevention of stress signaling upon exposure to a cold stimulus is crucial to safe lowering of metabolism, underscoring the need for validation of the R_x_—(S)—Z_tn_ relationship. This would require knowledge on both the location and function of the Z_tn_, which, as alluded to previously, is currently beyond our reach. However, by investigating the impact of a stimulus such as T_a_ or R_x_ on thermogenic effectors, heat loss effectors, and behavioral thermoregulation (Z_tn_—T_b_, Figure 3B), the Z_tn_ issues can be circumvented while still gaining insight into the Z_tn_ lowering potency. Z_tn_-related research is presently conducted in this fashion, whereby ancillary parameters (effectors and behavior) are used as a gold standard to gauge Z_tn_ [58].

Due to their high T_a_—T_b_ convective efficiency, small animals constitute ideal subjects for screening the anapyrexic potential of an R_x_ or investigating the effects of hypoxia. In larger animals, the lower T_b_—T_a_ convective efficiency necessitates the use of active cooling to accommodate induction of hypothermia and corollary hypometabolism. If active cooling in larger animal models is omitted, an anapyrexic agent or hypoxia may yield hypometabolic results in small animals but induce limited or no effect in larger animals. A totally T_b_-independent R_x_ (i.e., R_x_— Q, T_b_ = 37 °C) would be in contradiction to this model and in fact disprove the necessity of T_a_—T_b_ convection. It is our opinion, however, that hypometabolism cannot occur under normothermic conditions – an opinion that is supported by extension of the Arrhenius law.

When translating these principles to a clinical setting, the use of the R_x_(—S)—Z_tn_ relationship suggests that better outcomes would be achieved if hypothermic patients were pretreated with an anapyrexic agent (R_x_—Z_tn_—T_b_(—T_a_)) or subjected to hypoxia (S—Z_tn_—T_b_(—T_a_)). The fact that clinical practice deviates from these approaches may contribute to the increased comorbidity in patients as a result of hypothermia- inflicted stress responses during trauma-induced and perioperative hypothermia [15,162]. Presently, none of the strategies aimed to resolve these responses in patients encompass guidelines for Z_tn_ modulation. As a result, many patients are placed on 100% O_2_ and symptomatic treatment of shivering without a clear rationale. According to our model, a more cautious approach in oxygenating hypothermic patients could be beneficial, as reflected by the S—Z_tn_—T_b_ signaling axis. By subjecting a hypothermic patient to a hypoxic signal (S—Z_tn_) or anapyrexic agent (R_x_(—S)—Z_tn_), the cold-induced stress response may be mitigated by reduction of Z_tn_ through CB signaling and alignment of T_b_ with T_a_. As exposure to a cold stimulus readily activates thermal effectors such as shivering and BAT[163,164], such an effect would be promptly visible. However, to date no anapyrexic agents or hypoxic signaling mechanisms have been reported in a clinical setting.

## 3. Conclusions

In conclusion, the lack of understanding of the induction mechanisms underlying natural hibernation stands in the way of successful application of artificial hibernation in biotechnology and medicine. Accordingly, a model was developed to assist in finding the means to translate the physiological changes observed during natural hibernation to its artificial counterpart. Summarized in Figure 11, six essential elements form the basis of our model, which were extrapolated from literature. The relationships between these elements dictate their values and collectively govern the induction and sustenance of a hypometabolic state. To illustrate the potential validity of this model, various R_x_ (HIT, DOP, H_2_S, 5’-AMP, TAM, 2-DG, Mg^2+^) were described in terms of their influence on the intervariable relationships and effects on Q.

Although the ultimate purpose of this hypothetical model was to help expand the paradigm regarding the mechanisms of hibernation from a physiological perspective and to assist in translating this natural phenomenon to the clinical setting, our model only comprises a part of the vastly complex biological systems that underlie anapyrexia and hypometabolism. Moreover, readers should note that concepts as ‘set point’ are model-based phenomena, rather than neurobiological constructs. The key to the mechanistic underpinning of anapyrexic signaling currently rests on the shoulders of neurobiology, which is slowly unveiling the neurological signaling pathways. In that respect, thermal reflexes (cold defense, fever, anapyrexia, hibernation, etc.) are mediated by changes in the discharge of neurons in neural circuits controlling thermoregulatory effectors, and understanding how and through which neurochemical mediators these reflexes are effected will only be accomplished through detailed neurobiological experimentation. Accordingly, basic elucidation of the neurochemistry of anapyrexia is needed for the identification of useful Rxs [81, 82].

## Abbreviations:

2-DG: 2-deoxyglucose
5’-AMP: 5’-adenosine monophosphate
A_1_AR: A_1_ adenosine receptor
A: anabolism
BAT: brown adipose tissue
C: catabolism
DOP: delta-opioid
H_2_S: hydrogen sulfide
POAH: preoptic anterior hypohalamus
Q: metabolism
Rx: pharmacological agent
S: substrate
TAM: thyronamine
T_a_: ambient temperature
T_b_: core body temperature
Z_tn_: thermoneutral zone
